# Reliability and validity of a clinical competence test for dietitians caring patients with early chronic kidney disease

**DOI:** 10.1017/jns.2022.4

**Published:** 2022-02-15

**Authors:** Roxana M. Márquez-Herrera, Laura Cortés-Sanabria, Alfonso M. Cueto-Manzano, Héctor R. Martínez-Ramírez, Enrique Rojas-Campos, Claudia N. Orozco-González, Aaron González-Palacios

**Affiliations:** 1 Centro Universitario de Ciencias de la Salud, Universidad de Guadalajara, Sierra Mojada No. 950, Guadalajara, Jalisco, México; 2 Unidad de Investigación Médica en Enfermedades Renales, Hospital de Especialidades, Instituto Mexicano del Seguro Social, Belisario Dominguez No. 1000, Guadalajara, Jalisco, México; 3 Universidad Internacional Iberoamericana, Calle 15 No. 36, Campeche, México.

**Keywords:** Chronic kidney disease, Clinical competence, Dietitian, Reliability, Validity, CC, clinical competence, CKD, chronic kidney disease

## Abstract

The aim of the present study was to develop and validate a test to evaluate dietitian's clinical competence (CC) about nutritional care in patients with early chronic kidney disease (CKD). The study was conducted through five steps: (1) CC and its dimensions were defined; (2) test items were elaborated, and choice of response format and scoring system was selected; (3) content and face validity were established; (4) test was subjected to a pilot test and those items with inadequate performance were removed; (5) criterion validity and internal consistency for final validation were established. A 120-items test was developed and applied to 207 dietitians for validation. Dietitians with previous CKD training obtained higher scores than those with no training, confirming the test validity criterion. According to item analysis, Cronbach's *α* was 0⋅85, difficulty index 0⋅61 ± 0⋅22, discrimination index 0⋅26 ± 0⋅15 and inter-item correlation 0⋅19 ± 0⋅11, displaying adequate internal consistency.

## Introduction

Chronic kidney disease (CKD) is a serious public health issue worldwide; the global estimated prevalence is 11–13 %, with the majority of cases at early stages^(1)^. Strategies to reduce burden and costs related to CKD include the prevention and management of those patients at risk or with early renal function decline^(2)^.

Appropriate nutritional care has been recognised as a key factor in this endeavour. Dietitians can help to manage early CKD and prevent CKD onset in high-risk groups (i.e. diabetes mellitus and hypertension) by providing dietary advice to weight reduction, decrease blood pressure, glycaemic control, improve lipid profile, reduction of microalbuminuria/proteinuria and provide advice on healthy eating strategies^(3,4)^. Nevertheless, gaps in knowledge regarding CKD prevention and early treatment in high-risk groups were reported among these health professionals. A previous study showed that only 23 % of dietitians self-reported having adequate knowledge to provide nutritional care in early CKD; furthermore, solely 10 % of them identified diet contribution to initial kidney damage^(5)^.

In order to provide a complete, safe and suitable care as part of multidisciplinary team, it is crucial that dietitians comprehend the role of nutrition management in the prevention and early treatment of CKD^(6)^; therefore, they need to know how to implement knowledge, i.e. possess clinical competence (CC), which combines knowledge, skills and attitudes in clinical situations^(7)^. Educational interventions designed to increase CC of family physicians in the management of early CKD already have demonstrated a positive effect on kidney function; however, variables related to healthy eating and lifestyle need the intervention of a qualified dietitian to help delay CKD progression^(8)^.

A first step to measure and improve CC among dietitians, particularly in primary health care, is having an adequate instrument to determine it and identify knowledge gaps for a better tailoring of resources. There are no studies determining the CC of dietitians with regard to improving patients’ kidney function and preventing kidney disease progression. It is necessary to develop a tool to assess the skill of acquiring information from a variety of human and laboratory sources, to analyse and interpret these data, and finally to translate such findings into a rational diagnostic and/or management plan^(7)^. Therefore, the aim of the present study was to develop and validate a test to evaluate dietitian's CC about nutritional care of patients with early CKD.

## Subjects and methods

### Study design

**Fig. 1.**
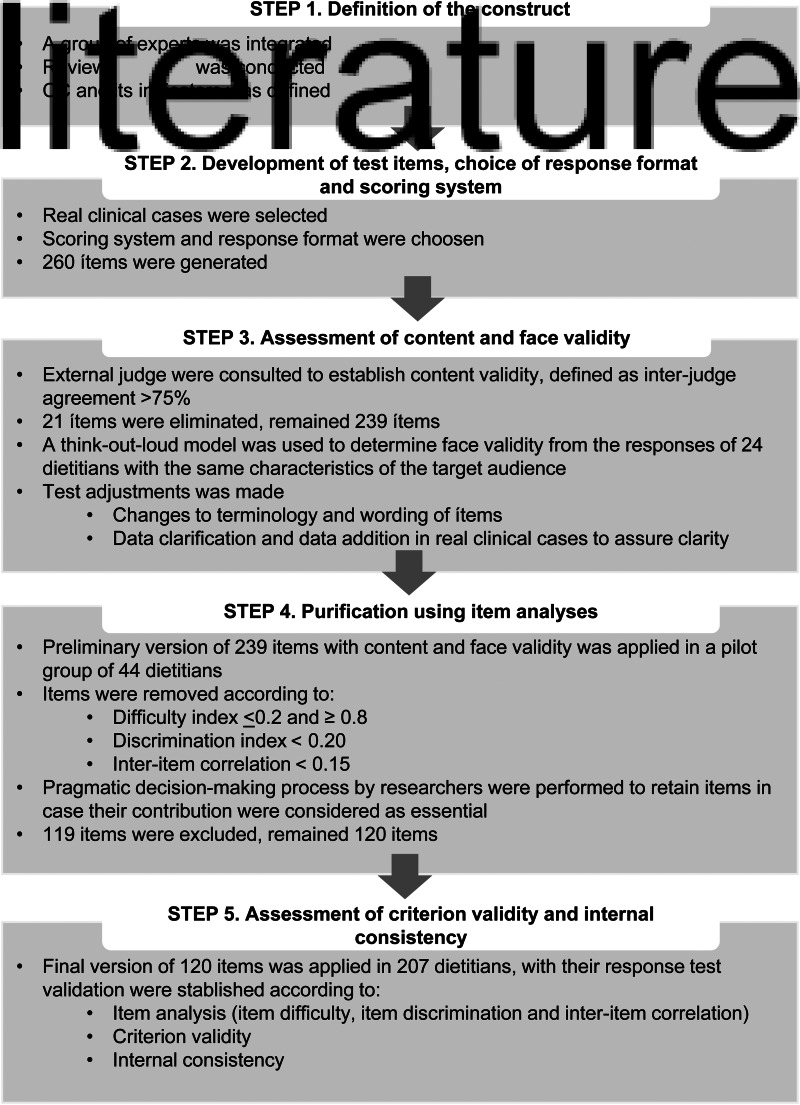
Steps involved to develop and validate the test to evaluate dietitian's clinical competence about nutritional care of patients with early CKD.

A descriptive validation study was carried out. The test development and validation consisted of five steps: steps 1 and 2 included the process of initial test development to measure CC about early CKD among dietitians, steps 3 and 4 lead to a preliminary test that finally was subjected to validation in step 5 (Fig. 1). Methodology to develop and evaluate nutrition knowledge questionnaire proposed by Trakman *et al.*^(9)^ was used as a guide to achieve the objective of the present study.

#### Step 1. Definition of the construct

The first step was to define CC regarding nutrition in early CKD to ensure an appropriate conceptual framework. For this purpose, a workshop was held with a group of seven experts: three renal dietitians, three physicians (nephrology, internal medicine and family medicine) and one psychometrics expert. According to Miller^(7)^, health professionals must know how to use the knowledge they have accumulated, and develop, among other things, the skill of acquiring information from a variety of sources, to analyse and interpret the data, and finally to translate such findings into a rational diagnostic or management plan. On the basis of this construct, CC was defined as the ‘ability to integrate and apply knowledge, skills and judgement to provide safe and effective nutritional care in CKD’. Competence was evaluated using the following indicators: identification of risk factors for CKD, diagnosis integration, adequate use of therapeutic resources and iatrogenesis recognition, as described in Table 1.

**Table 1.** Definition of clinical competence and its indicators

CC indicator	Definition
Identification of risk factors for CKD	Distinguish a condition, behaviour or another factor, that increases risk or susceptibility to the development or progression of CKD, for example, high blood pressure, hyperglycaemia, male sex and age, among others.
Diagnosis integration	The process of identifying nutritional problems, as well as the nature of CKD. This process involves information gathering from health and food history, physical exam, biochemical tests and clinical reasoning to make a statement or conclusion from such data.
Adequate use of therapeutic resources	Specific preventive, therapeutic or follow-up activities, and recommendations that are the most effective for patients, with few undesirable effects and clear potential benefits.
Iatrogenesis recognition	Identifying certain actions that should be made by the health professional to avoid harming the patient or actions that are not justified, or do not reward patients in terms of well-being, and can even cause harm.

#### Step 2. Development of test items, choice of response format and scoring system

Once CC and its indicators were defined, in the same workshop, four clinical cases from patients at risk or with early CKD at the primary healthcare were selected. By using these real clinical cases, and considering the conceptual framework developed in Step 1, as well as relevant reviewed literature (including clinical practice guidelines and peer-reviewed journal articles), items (close-ended questions) were generated by the panel of experts. Two hundred sixty items were initially generated, following the suggestion to develop an item bank with at least double the amount that will appear in the final version^(10)^ (about 100 final items were expected; twenty-five per each clinical case). Answer options were true, false and don't know. A balance between true and false statements (50 % of each option) was achieved. The scoring system was as follows: each correct answer adds one point (+1), incorrect answer subtracts one point (−1) and don't know option did not affect the score. The balance between true and false statements, and the ‘don't know’ option, may reduce the probability of correctly guessing the correct option, as previously suggested^(10)^.

#### Step 3. Assessment of content and face validity

Content validity relates to the ability of a questionnaire to adequately cover all relevant topics of the construct^(11)^. To assure content validity, the initial test (260 items) was evaluated by external judges regarding relevance, a group of three to ten experts is required to meet this goal^(12)^; in the present study, four dietitians specialised in nephrology (different from those of the group of experts constructing the test), and recognised as experts in our setting, were the external judges. Each external judge rated individual items for relevance on a 4-point Likert scale (1 = not relevant, 2 = somewhat relevant, 3 = relevant, 4 = very relevant). The content validity index was calculated by dividing the number of external judges who scored the item as 3 or 4 divided by the total number of judges; a score of >0⋅75 was considered as adequate to keep an item^(12)^. After this evaluation, from the initial 260 items, twenty-one were eliminated, remaining 239 items.

Face validity refers to the appearance of reasonableness of the test from the perspective of the test-taker and could be considered an aspect of content validity^(11)^. Consequently, the test was completed by twenty-four dietitians with the same characteristics of the target audience for whom it was created, in order to confirm that instructions were easy to follow, to determine how long the test requires to complete and to evaluate the appropriateness and formulation of the items. A think-out-loud model was used, in which the participants verbalise their thought process as they complete the test, and when finalised the whole test, they meet with the researcher to discuss his/her experience of completing it. After this process, the researcher made necessary changes to terminology, wording and adding specific data to ensure face validity.

#### Step 4. Purification using item analysis

Once results of content and face validity processes were incorporated into the test, a preliminary version of 239 items was applied to a pilot group of forty-four dietitians with similar characteristics of the target sample to perform item analysis. Item analysis refers to a range of classical test theory techniques based on mathematically correlations between items and the degree that a person's true score on a test reflects their observed score and measurement error^(9)^. For this purpose, results of the pilot group item difficulty, item discrimination and inter-item correlation were analysed and used to eliminate those items with inadequate performance.

- Item difficulty (severity or facility) assesses the percentage of respondents correctly answering an individual item^(10)^. For example, if 90 % of participants answer correctly a particular item, the difficulty index was calculated as 0⋅9. Items with difficulty indexes of <0⋅2 and >0⋅8 were considered as too difficult and too easy, respectively, and were excluded^(10)^.
- Item discrimination is calculated based on the general notion that high-scoring students tend to choose the right answer and low-scoring students tend to choose the wrong answer^(10)^. If a person does well overall but poorly on a particular item (and vice versa), the item is said to be a poor judge (or discriminator) of knowledge^(9)^. To estimate the discrimination index, cut-off 25th and 75th percentiles of the total score were used, then the percentage of individuals in each group who endorsed correct/incorrect statements was evaluated^(13)^. For example, if 70 % of the high-scoring group and 20 % of the low-scoring group endorse a particular item in the scale, the discrimination index for that item was calculated as 0⋅75−0⋅20 = 0⋅55. Items with a discrimination index of <0⋅20 were removed^(10)^.
- Inter-item correlation exposes that items with very high correlations may be assessing the same idea, whereas items with low correlations may suggest items that are too assorted to be assessing an isolated concept^(9)^. For example, an item with inter-item correlation of 0⋅95 can be considered redundancy among the items, whereas an item with inter-item correlation of 0⋅1 is assessing a very different idea from the rest of the items. From this analysis, those items with inter-item correlation coefficient of <0⋅15 were eliminated^(14)^.

In spite of results of item analysis, some individual items were useful beyond their contribution to the total score and provided key information about gaps in CC about early CKD among dietitians. Therefore, a pragmatic decision-making process before removing items was employed, as previously suggested^(15)^. At the end of this step, 119 items out of 239 of the preliminary test were excluded. The final test contained 120 items (available as supplementary material).

#### Step 5. Assessment of criterion validity and internal consistency

The final test (120 items) was applied to 207 dietitians for the assessment of internal reliability and criterion validity and compute the final item analysis on a large sample.

Criterion validity refers to the degree to which scores of the test are an adequate reflection of a ‘gold standard’; in the present study, it was expected that dietitians with previous training had the highest mean score, which differs significantly to demonstrate criterion validity.

Internal consistency assesses the degree to which items within a test are interrelated and measures the same construct^(11)^. Cronbach's *α* was used to determine internal consistency.

### Recruitment and study sample

The recruitment of dietitians was done by means of the ‘snowball technique’, in which one interviewee gives the researcher the name of at least one more potential interviewee that met the inclusion criteria for the study^(16)^, meaning professional with a bachelor's degree in nutrition complete, graduates from the University of Guadalajara, the largest university in our setting (Jalisco, México), or from a university incorporated (same academic curricula) to the last. Invitations were sent, and for those who agreed to participate, the test application was individual or in an organised meeting with small groups. The recruitment and data collection period was January 2018–January 2019.

### Ethical considerations

This study was conducted according to the guidelines laid down in the Declaration of Helsinki and all procedures involving research study participants were approved by the Ethics Committee of the University of Guadalajara, under protocol number 01917. Written informed consent was obtained from all subjects/patients.

### Statistical analysis

Data are shown as mean ± standard deviation or median and 25th–75th percentiles, when dimensional variables had parametric or non-parametric distribution, respectively, or as percentage in the case of nominal variables.

For test validation (step 5), the following analyses and cut-offs were used.

For internal consistency, a Cronbach's *α* of ≥0⋅70 was considered adequate^(9)^. To establish criterion validity, the Mann–Whitney *U* test was applied to compare dietitians with and without previous CKD training. The latter test and *χ*^2^ were used to compare general characteristics between both groups of dietitians. A *P* value of <0⋅05 was considered as significant.

Items with difficulty index in the range of 0⋅2–0⋅8, discrimination index >0⋅2, inter-item correlation >0⋅15 and balance of 50 and 50 % of true and false statements were considered as ‘ideal’, as previously reported^(9,10,14,17)^. Tests with missing data were not included in the analysis.

## Results

Two hundred and seven dietitians participated in test validation of the final version containing 120 items (Table 2). Most participants were women (91 %), aged 26 (24–29) years. A majority had a bachelor's degree, only 9 % had a master's degree or PhD and the median time after graduation was 4 (1–6) years. Participants had 2 (0⋅5–3) years of practice in nutrition counselling, and 38 % treated patients with CKD. Forty-eight percent reported had previous training in non-communicable diseases (i.e., diabetes, hypertension, dyslipidaemia and obesity), and 20 % had previous CKD training; the latter subjects were considered as the ‘gold standard’ group to establish criterion validity. Table 2 also shows comparisons of characteristics of dietitians with and without CKD training. Participants with previous CKD training had significantly more years of laboral experience, treated more patients with CKD and had training in other non-communicable diseases more frequently than those without previous CKD training.

**Table 2.** Characteristics of dietitians in the final test (120 items), according to the previous CKD training

Characteristic	All *n* 207	Without CKD training *n* 166	With CKD training *n* 41	*P* value
Female sex, *N* (%)	188 (91)	149 (90)	39 (95)	0⋅377
Age, years	26 (24–29)	26 (24–29)	27 (24–29)	0⋅308
Academic degree, %				
Bachelor's degree	189 (91)	154 (93)	35 (85)	0⋅118
Master's degree or PhD	18 (9)	12 (7)	6 (15)	
Laboral experience, years	4 (1–6)	4 (1–5)	5 (2–7⋅5)	0⋅030
Nutrition counselling practice, years	2 (0⋅5–3)	2 (0⋅5–3)	2⋅5 (1–6)	0⋅069
Main workplace, *N* (%)				
Public health institution	53 (26)	40 (24)	13 (32)	
Private practice	90 (44)	72 (44)	18 (44)	0⋅741
Other	61 (30)	51 (32)	10 (24)	
Treating CKD patients, *N* (%)	79 (38)	51 (31)	28 (68)	<0⋅0001
Training in other non-communicable disease	99 (48)	68 (41)	31 (76)	<0⋅0001

PhD, philosophy degree; CKD, chronic kidney disease; CC, clinical competence.

Data are shown as percentage or median (25th–75th percentiles).

From responses of the 207 dietitians subjected to the final test, the item analysis was as follows: 73 % had a difficulty index between the average (0⋅2–0⋅8), 2⋅5 % was classified as difficult and 24 % as easy. In addition, 62 % of the items had an adequate discrimination index (above 0⋅20), and 67 % had an adequate inter-item correlation (above 0⋅15). Table 3 shows the item analysis and internal consistency grouped according to CC indicators. The items belonging to each CC indicator had a balance near to 50 % between true and false statements. Mean values of difficulty index, discrimination index and inter-item correlation were as recommended in all groups of items, except for the discrimination index of identification of risk factors for CKD. Regarding Cronbach's *α* coefficient, the value of the complete test was 0⋅85, considered as an appropriate internal consistency to evaluate global CC, whereas the *α* values of the individual CC indicators scored lower.

**Table 3.** Item analysis of the final test (120 items)

CC indicator	Number of items	Balance between true and false answers		Difficulty index		Discrimination index		Inter-item correlation		Cronbach's *α*
*Ideal value*	NA	‘True’ statements 50 %	‘False’ statements 50 %	0⋅2–0⋅8		≥0⋅20		≥0⋅15		≥0⋅70
				Mean	SD	Mean	SD	Mean	SD	
Identification of risk factors for CKD	14	6 (43 %)	8 (57 %)	0⋅50	0.19	0⋅18	0.16	0⋅15	0.12	0⋅54
Diagnosis integration	40	17 (43 %)	23 (57 %)	0⋅60	0.24	0⋅28	0.15	0⋅20	0.11	0⋅66
Adequate use of therapeutic resources	32	17 (53%)	15 (47%)	0.67	0.23	0.26	0.12	0.20	0.10	0.63
Iatrogenesis recognition	34	14 (41 %)	20 (59 %)	0⋅63	0.18	0⋅29	0.14	0⋅21	0.10	0⋅66
Global CC (whole test)	120	54 (45 %)	66 (55 %)	0⋅61	0.22	0⋅26	0.15	0⋅19	0.11	0⋅85

NA, not applicable; CC, clinical competence; CKD, chronic kidney disease; SD, standard deviation.

Data are shown as mean ± standard deviation.

Table 4 displays the results of assessment of criterion validity according to the previous CKD training status. As expected, the mean score of the global CC as well as the individual CC indicators were significantly higher for dietitians with CKD training in comparison with dietitians without previous training, supporting the criterion validity.

**Table 4.** Assessment of criterion validity by mean score comparison according to the previous CKD training status

CC indicator	Without CKD training *n* 166	With CKD training *n* 41	*P* value
Identification of risk factors for CKD	3 (1–4)	5 (2–7)	0⋅001
Diagnosis integration	13 (8–17)	15 (10–22)	0⋅04
Adequate use of therapeutic resources	14 (11–18)	19 (14–22)	0⋅001
Iatrogenesis recognition	12 (7–17)	18 (12–22)	<0⋅0001
Global CC (whole test)	42 (29–52)	58 (43–70)	<0⋅0001

CC, clinical competence; CKD, chronic kidney disease.

Data are shown as median (25th–75th percentiles).

## Discussion

The present study was performed to develop and validate a test evaluating dietitian CC about nutritional care in patients with early CKD. Nutrition intervention is effective in the control of risk factors for CKD but demands specialised training^(6,18)^; noteworthy, there is a scarcity of tools in this area.

Validation was achieved in several aspects. First, the availability of some experienced dietitians made it possible to ensure content and face validity (items covered the concepts they intend to measure and have clear instructions and adequate vocabulary). Additionally, purification of items, eliminating those redundant or inappropriate, reduced size of the test at the time that improved performance. Secondly, the analysis of the final test results shows that most of the items had satisfactory difficulty, discrimination capacity and inter-item correlation while maintaining the balance of true/false statements. Thirdly, the complete test shows adequate internal consistency according to Cronbach's *α* coefficient; however, isolated CC indicators (test subsections) should be used cautiously as its *α* values decreased when analysed individually, previous reports suggest that a higher number of items is associated to greater internal consistency^(14,19)^. Finally, when test responses were compared between two groups of people who *a priori* were expected to have different results, results confirmed the criterion validity. Dietitians who had previous CKD training (selected as the ‘gold standard’ group) also had more training in other chronic diseases, more interaction with CKD patients and longer time after graduation compared to the group of dietitians who were expected to score worse on the test; these differences also contribute to ensure criterion validity.

The present study does have limitations and strengths. The sample size was not calculated *a priori* by probabilistic methods (methods for sample size calculation in validation studies are limited); however, some authors recognise that a sample size of ‘200’ is fair^(15)^ or satisfactory if include a greater number of respondents than the number of questions^(20)^, as done in the present study. Our sample was mainly composed of women; however, in our country (as in some others), women largely predominate as nutrition professionals^(21)^. On the other hand, conditions to evaluate the scale's factor structure using exploratory factor analysis were not met, nor the evaluation of temporal stability using test–retest. The latter would be related to the length of the test (more than 2 h to complete) that could limit the recruitment of health workers. Notwithstanding, except for the latter analyses, the validation in the present study included all other steps of the methodology for a nutrition knowledge questionnaire^(9)^: definition of the construct by a panel of experts, generation of the item pool, choice of an appropriate scoring system and response format, assessment of content and face validity, purification of the scale and gathering of data to re-examine the questionnaire's properties considering item difficulty, item discrimination, inter-item correlation, internal consistency and criterion validity. Adherence to this rigorous test development and validation process is one of the strengths of the present study, but the main merit might be to have evaluated CC, because this involves assessing not only knowledge, but also its application, and the capability of dietitians to identify risk factors for CKD, integrate a diagnosis, use appropriately therapeutic resources and recognise iatrogenic behaviour. In clinical setting, all those aspects are relevant since lack of training can negatively affect the nutritional therapy of patients on risk to progress to end stages of CKD. A previous test assessing CC in early CKD was validated for primary care physicians^(8)^, but not for dietitians. To the best of our knowledge, the present is the first test developed in this regard.

### Practical implications

CKD is a growing epidemic worldwide, and dietitians have an essential role in its prevention and management; however, it is first necessary that dietitians display adequate knowledge and CC in this regard. It is recognised that the use of poor-quality nutrition knowledge questionnaires with unknown validity and/or reliability limits the conclusions that can be drawn from research in the nutrition education field^(20)^. The present questionnaire describes step by step, in a detailed manner, the process of development and validation to assess dietitians’ CC, and it is a suitable and easy tool to differentiate professionals with adequate competence from those who require further training. Moreover, the use of this test could reliably identify the effect of educational interventions to improve CC and catalyse investigation on dietitian education.

## Conclusions

In conclusion, the present study encompassed the development and validation of a test to determine dietitian's CC about nutritional care of patients with early CKD. The final test has face, content and criterion validities, as well as satisfactory internal consistency.

The developed test is a reliable and valid tool to assess dietitian CC about nutritional care of patients with early CKD. It is a promising test in the development and guidance of public health actions related to the prevention and early management of CKD patients. Research is warranted to determine the CC of early CKD among dietitians.

## Supplementary material

For supplementary material accompanying this paper visit https://doi.org/10.1017/jns.2022.4. click here to view supplementary material

## References

[1] Hill N, Fatoba S, Oke J, (2016) Global prevalence of chronic kidney disease – a systematic review and meta-analysis. PLoS ONE 11, e0158765. 27383068 10.1371/journal.pone.0158765 PMC4934905

[2] Obrador G, Mahdavi-Mazdeh M & Collins A (2011) Global kidney disease prevention network: a position statement from the national kidney foundation. Am J Kidney Dis 57, 361–370. 21335246 10.1053/j.ajkd.2010.12.006

[3] Kramer H (2019) Diet and chronic kidney disease. Adv Nutr 10, S367–S379. 31728497 10.1093/advances/nmz011 PMC6855949

[4] Ikizler TA, Burrowes JD, Byham-gray LD, (2020) KDOQI clinical practice guideline for nutrition in CKD: 2020 update. Am J Kidney Dis 76, S1–107. 32829751 10.1053/j.ajkd.2020.05.006

[5] Márquez-Herrera R, Cueto-Manzano A & Cortés-Sanabria L (2017) Role of dietitian in prevention and treatment of early chronic kidney disease. Rev Med Inst Mex Seguro Soc 55, S175–S181. 29697239

[6] Anderson CAM & Nguyen HA (2018) Nutrition education in the care of patients with chronic kidney disease and end-stage renal disease. Semin Dial 31, 115–121. 29455475 10.1111/sdi.12681

[7] Miller G (1990) The assessment of clinical skills/competence/performance. J Assoc Am Med Coll 65, S63–S67.

[8] Cortés-Sanabria L, Cabrera-Pivaral CE, Cueto-Manzano AM, (2008) Improving care of patients with diabetes and CKD: a pilot study for a cluster-randomized trial. Am J Kidney Dis 51, 777–788. 18436088 10.1053/j.ajkd.2007.12.039

[9] Trakman GL, Forsyth A, Hoye R, (2017) Developing and validating a nutrition knowledge questionnaire: key methods and considerations. Public Health Nutr 20, 2670–2679. 28735598 10.1017/S1368980017001471 PMC10261290

[10] Haladyna T (2004) Developing and Validating Multiple-Choice Test Items, 3rd ed. Mahwah, NJ: Lawrence Erlbaum Associates, Inc.

[11] Mokkink LB, Terwee CB, Patrick DL, (2010) The COSMIN study reached international consensus on taxonomy, terminology, and definitions of measurement properties for health-related patient-reported outcomes. J Clin Epidemiol 63, 737–745. 20494804 10.1016/j.jclinepi.2010.02.006

[12] Polit D, Tatano Beck C & Owen S (2007) Is the CVI and acceptable indicator for content validity? Appraisal and recommendations. Res Nurs Health 30, 459–467. 17654487 10.1002/nur.20199

[13] Cappeleri J, Lundy J & Hays R (2014) Overview of classical test theory and item response theory for quantitative assessment of items in developing patient-reported outcome measures. Clin Ther 36, 648–662. 24811753 10.1016/j.clinthera.2014.04.006 PMC4096146

[14] Streiner DL (2003) Starting at the beginning: an introduction to coefficient alpha and internal consistency. J Pers Assess 80, 99–103. 12584072 10.1207/S15327752JPA8001_18

[15] DeVellis R (2016) Scale Development: Theory and Applications, 4th ed. New York: SAGE Publications, Inc.

[16] Kirchherr J & Charles K (2018) Enhancing the sample diversity of snowball samples: recommendations from a research project on anti-dam movements in Southeast Asia. PLoS ONE 13, 1. 10.1371/journal.pone.0201710 PMC6104950 30133457

[17] Hingorjo MR & Jaleel F (2012) Analysis of one-best MCQs: the difficulty index, discrimination index and distractor efficiency. J Pak Med Assoc 62, 142–147. 22755376

[18] Kent PS, McCarthy MP, Burrowes JD, (2014) Academy of nutrition and dietetics and national kidney foundation: revised 2014 standards of practice and standards of professional performance for registered dietitian nutritionists (competent, proficient, and expert) in nephrology nutrition. J Acad Nutr Diet 114, 1448–1457. 25169785 10.1016/j.jand.2014.05.006

[19] Steyn NP, Labadarios D, Nel JH, (2005) Development and validation of a questionnaire to test knowledge and practices of dietitians regarding dietary supplements. Nutrition 21, 51–58. 15661478 10.1016/j.nut.2004.09.008

[20] Parmenter K & Wardle J (2000) Evaluation and design of nutrition knowledge measures. J Nutr Educ 32, 269–277.

[21] King JC (2003) Contributions of woman to human nutrition. J Nutr 133, 3693–3697. 14608097 10.1093/jn/133.11.3693

