# Supplementary material for: Reliability and validity of a clinical competence test for dietitians caring patients with early chronic kidney disease
Source: J Nutr Sci. 2022 Feb 15;11:e10. doi: 10.1017/jns.2022.4 (PMC8889087; doi:10.1017/jns.2022.4)
Supplement: Supplementary file 1 [file S2048679022000040sup001.docx]

**INSTRUMENTO PARA MEDIR LA COMPETENCIA PROFESIONAL DEL NUTRIÓLOGO EN ATENCIÓN AL PACIENTE CON ENFERMEDAD RENAL CRÓNICA TEMPRANA**

Nombre: ___________________________________________________________________

Fecha: _____/_____/____

**INSTRUCCIONES**

A continuación usted encontrará cuatro casos clínicos reales. Después de cada caso están las preguntas, que se distinguen por estar marcadas con letras mayúsculas en negrillas, tener unas palabras claves subrayadas y después de dos puntos, van seguidas de enunciados marcados con numeración arábiga continua que complementan la pregunta. Usted deberá dar respuesta a cada enunciado (un total de 120); para ello deberá consultar el glosario que contiene las palabras clave. Es muy importante que al decidir su respuesta lo haga desde lo recomendado idealmente, aunque no cuente con todo lo necesario para ejercer su práctica.

Usted deberá anotar la respuesta sobre la línea izquierda que aparece antes de cada enunciado. Para ello debe leer la pregunta, atender el significado de las palabras subrayadas, así como la totalidad del enunciado para optar por una de las siguientes respuestas: verdadero, falso o no sé.

- Contestar VERDADERO (V) si usted considera que la pregunta con sus palabras clave y el enunciado **corresponde** a lo que explícitamente se afirma en conjunto. Por ejemplo, “sí es un factor de riesgo”, “es un diagnóstico PROBABLE”, “es o fue una aplicación JUSTIFICADA de recursos diagnósticos y terapéuticos”, etc.
- Contestar FALSO (F) si usted considera que la pregunta con sus palabras clave y el enunciado **no corresponde** a los que explícitamente se afirma en conjunto. Por ejemplo, “no es un factor de riesgo”, “es un diagnóstico IMPROBABLE”, “es o fue una aplicación INJUSTIFICADA de recursos diagnósticos y terapéuticos”, etc.
- Contestar NO SÉ (NS) si usted no puede decidir entre verdadero y falso.

**GLOSARIO DE PALABRAS CLAVE PARA DECIDIR LA RESPUESTA**

Factor de riesgo: Se refiere a la existencia demostrada de antecedentes o condiciones actuales del paciente, que sin formar parte de un padecimiento, hacen más probable su aparición o que éste se agrave.

Compatible: Se refiere al hecho de que la información recabada acerca de un paciente concuerda con la entidad o entidades propuestas como posibles diagnósticos.

A favor: Presencia de un dato clínico, de laboratorio o de gabinete, que forma parte y es base importante en el diagnóstico de la enfermedad referida en la pregunta.

Útiles: Hace referencia a ciertos procedimientos o medidas de diagnóstico que presentan claramente más beneficios que perjuicios para el paciente.

Apropiadas: Hace referencia a ciertas medidas o recomendaciones preventivas, terapéuticas o de seguimiento, que son las más efectivas para el paciente, con escasos efectos indeseables y claros beneficios inmediatos, mediatos o potenciales.

Necesarias: Hace referencia a ciertas acciones que debió realizar el profesional de la salud para evitar daño al paciente (desde el daño leve hasta el grave, a veces la muerte).

Innecesarias: Hace referencia a ciertas acciones de parte del profesional de la salud que no están justificadas para el paciente, no le retribuyen en bienestar y además pueden llegar a producirle daño.

A continuación se muestra un caso clínico como ejemplo de cómo responder cada enunciado (no incluye todos sus enunciados, los que aparecen tienen su número original):

Niña de una semana de edad traída por la madre de 22 años, ama de casa, con secundaria completa; padre de 23 años, chofer de una fábrica. Hermano de 1 año 3 meses. Producto de un segundo embarazo de 40 semanas, control prenatal adecuado. Al nacer, tuvo llanto y respiración espontáneos, pesó 3.2 Kg. Alimentación principal con fórmula láctea maternizada en dilución 1:1. La madre está preocupada porque en las últimas tres evacuaciones han tenido moco, grumos blancos y puja mucho; decidió darle té de manzanilla porque piensa que está empachada. Peso 3.4 Kg, talla 53 cm, perimetro cefálico 33 cm, FC 120xminuto, FR 26xminuto, temperatura 37ºC. Tranquila, fontanela normotensa, ictericia +/++++ en tegumentos, abdomen blando con cicatriz umbilical normal. Despierta y llora con lágrimas. Se le recomendó alimentación exclusiva al seno materno y cita abierta.

**A)** En esta paciente son factores de riesgo para su inadecuado cuidado familiar:

1) F Escolaridad de la madre.

2) V Edad del hermano.

3) Etc.

El primer enunciado fue contestado correctamente porque el nivel de escolaridad que se considera como riesgo es primaria incompleta.

El segundo fue contestado correctamente porque el hermano requiere mucho cuidado por su edad.

**B**) Son datos a favor de una recién nacida con crecimiento normal:

9) F El peso.

La respuesta al noveno enunciado es incorrecta porque con el peso no se valora el crecimiento de un niño.

**C)** En esta paciente, son diagnósticos compatibles:

12) V Ictericia fisiológica del recién nacido

El doceavo enunciado es correcto porque por la edad y la presencia de ictericia, sin mas datos que refieran una patología indican ictericia fisiológica del recién nácido.

**D)** En esta paciente son útiles los siguientes indicadores para precisar el diagnóstico nutricio:

17) NS Perímetro cefálico.

El décimo séptimo enunciado fue contestado como NS porque la persona no pudo decidir entre verdadero y falso.

**E)** Eran acciones necesarias para valorar el estado nutricio de la paciente pero que nutrición no realizó:

19) V Preguntar los motivos porque la alimentación principal es con fórmula láctea.

El décimo noveno enunciado tiene una respuesta correcta porque por la edad de la paciente es importante conocer los motivos por los cuales no se alimenta sólo con leche materna.

**F)** En esta paciente fueron medidas apropiadas:

31) V Recomendar lactancia exclusiva al seno materno.

El trigésimo primer enunciado tiene una respuesta incorrecta porque para hacer esa recomendación es necesario conocer los motivos por los cuales se le alimenta con fórmula.

Por cada respuesta correcta usted tendrá un punto porque contestó verdadero a lo que es verdadero, o porque resolvió falso a lo que es falso. Cada respuesta incorrecta le restará un punto, ya sea porque contestó verdadero a lo que es falso, o bien, contestó falso a lo que es verdadero. La respuesta no sé no le da puntos, ni le resta.

Cada enunciado sin contestar le resta un punto.

**CASO 1**

**Antecedentes personales:** Mujer de 58 años de edad, casada, camarera de un hotel, con primaria completa. Tiene diabetes mellitus (DM) tipo 2 desde hace 17 años. Su madre fue diabética y desarrolló insuficiencia renal crónica terminal. La paciente sufrió hemorragia cerebral hace 12 años.

**Exploración física:**

- Tensión arterial: 130/70 mmHg
- Peso: 54 kg, Talla 1.50 m, IMC 24 kg/m^2^
- El médico encontró hemiparestesia de extremidades izquierdas pero el resto de la exploración fue normal.

**Resultados de laboratorio**:

- Colesterol total: 274 mg/dL, Triglicéridos: 539 mg/dL
- Glucosa: 235 mg/dL
- Hemoglobina glucosilada: 10.6%
- Ácido úrico:5 mg/dL
- Fósforo 3.0 mg/dL
- Creatinina sérica: 0.9 mg/dL
- Tasa de filtración glomerular por CKD-EPI: 70 mL/min/1.73 m^2^
- Relación albúmina/creatinina: 116 mg/g
- Urianálisis: Glucosa 500 mg/dL

**Manejo del equipo de salud**.

En su cita con el ***médico*** refirió sudoración, disnea y cefalea continua; además ansiedad, fatiga y pérdida de energía casi a diario; negó poliuria, polidipsia y polifagia. Le indicó: insulina NPH 10 U por la noche y glibenclamida 5 mg 2 veces al día, bezafibrato 200 mg/día y se inició aspirina 500 mg/día y metoprolol 50 mg/día. Puesto que refirió ingierir dos refrescos regulares de cola al día, el médico le recomendó tomar un solo refresco por día, diluido en ½ vaso de agua y reducir el consumo de sal; cita en un mes con resultados de examen general de orina y glucosa sérica; se solicitó interconsulta a nutrición, enfermería, nefrólogía y psicología.

Al interrogatorio de la ***nutrióloga*** refirió que alguien le dijo que debía pesar 51 kg. Caminaba 30 minutos al día, dejó de hacerlo porque se agitaba al hablar, finalmente lo suspendió desde hace 1 mes por falta de tiempo. Al saberse diabética, dejó de cocinar con manteca y usa aceite vegetal de maíz. No le han indicado qué dieta seguir. Agrega azúcar al agua fresca y café; poca sal a sus platillos; hace tres comidas al día, con predominio de frijoles, huevo y carne; verduras 2 a 3 veces por semana, frutas a diario y aproximadamente 1½ L de agua al día.

Los resultados de la evaluación dietética fueron: Energía: 1650 kcal, con proteína 17% (1.3 g/kg peso actual), Hidratos de Carbono 56%, HC simples: 20%, Fibra 17 g, Grasas: 27%, Saturadas: 5%, Poli-insaturadas 17%, Monoinsaturadas: 5%

Se indicó un plan bajo en hidratos de carbono simples (< 10%), con reducción en proteínas (1.0 g/kg), incrementar consumo diario de verduras, así como realizar actividad física al menos 10 minutos diarios como meta inicial; consulta en un mes para nueva evaluación.

Le explicó a la ***enfermera*** que toma los medicamentos como le indica su médico pero ignora sus efectos secundarios. Reconoce los fármacos para la diabetes, así como los síntomas de hiperglucemia o hipoglucemia y sabe qué medidas tomar en su caso. No conoce las cifras de un control adecuado de glucosa, pero sí las complicaciones de la DM.

A la ***trabajadora social*** le comentó: convivir sólo con una vecina y ocasionalmente con sus parientes; tener comunicación deficiente con su esposo y buena con sus hijos; el ingreso familiar mensual es de 7,400.00 pesos, con lo que solventa gastos de vivienda, comida y servicios. Se le brindó soporte emocional y orientación sobre la importancia del tratamiento médico y del apoyo familiar; se indicó actividad física y reducir los hidratos de carbono.

1. **EN ESTA PACIENTE SON FACTORES DE RIESGO PARA ERC:**
2. ____ Nivel socioeconómico.
3. ____ Cifras de hemoglobina glucosilada.
4. ____ Cifras de tensión arterial.
5. **EL CUADRO DE ESTA PACIENTE ES COMPATIBLE CON:**
6. ____ Microalbuminuria.
7. ____ ERC estadio 1.
8. **EN ESTA PACIENTE SON DATOS A FAVOR DE ERC:**
9. ____ Cifra de glucosa en orina.
10. ____ Relación albúmina/creatinina en orina.
11. ____ Tasa de filtración glomerular y presencia de daño renal.
12. ____ Cifra de tensión arterial sistólica.
13. **EN ESTA PACIENTE SON INDICADORES ÚTILES PARA PRECISAR EL DIAGNÓSTICO NUTRICIO:**
14. ____ Pérdida de energía.
15. ____ Consumo de refrescos.

12) ____ Tensión arterial.

1. **DE ACUERDO CON EL ESTADO ACTUAL DE LA PACIENTE SON ACCIONES APROPIADAS QUE LA NUTRIÓLOGA HIZO:**
2. ____ Indagar sobre las técnicas de preparación de alimentos
3. ____ Reducir el consumo de proteínas.
4. ____ Disminuir el consumo de hidratos de carbono simples.
5. ____ Disminuir el consumo de grasas.
6. ____ Incrementar consumo de líquidos.

1. **ERAN ACCIONES NECESARIAS PARA EL ADECUADO MANEJO NUTRICIO DE ESTA PACIENTE, QUE LA NUTRIÓLOGA NO REALIZÓ:**
2. ____ Balancear el consumo de grasas.
3. ____ Disminuir el consumo de fósforo.
4. ____ Recomendar consumo de alfa cetoanálogos.
5. **FUERON ACCIONES INNECESARIAS PARA LA PACIENTE REALIZADAS POR EL PERSONAL EN SALUD:**
6. ____ Recomendar diluir el refresco en agua.
7. ____ Orientación sobre apego nutricio.
8. ____ Solicitar envío a psicología.
9. **EN LA PRÓXIMA CONSULTA DE ESTA PACIENTE LA NUTRIÓLOGA DEBE:**
10. ____ Ajustar la insulina de acuerdo sólo al resultado de su glucemia en ayuno.
11. ____ Ajustar la ingestión de líquidos según el resultado del EGO.
12. ____ Monitorizar la composición corporal.
13. ____ Incrementar actividad física a por lo menos 1 hora diaria.
14. ____ Sugerir que el médico solicite fracciones de colesterol y triglicéridos

en 3 meses.

**CASO 2**

**Antecedentes personales:** Hombre de 53 años, auxiliar de enfermería, casado, con DM tipo 2 e hipertensión arterial de 25 y 20 años de evolución respectivamente; fuma 20 cigarrillos por día desde hace 40 años. Hace 3 años fue tratado con láser por retinopatía en ambos ojos. Hace 1 mes su glucemia fue de 121 mg/dL, creatinina sérica de 1.0 mg/dL y albuminuria de 500 mg/g. Se refiere sin poliuria, polidipsia o polifagia; ha tenido dolor continuo, paresias y parestesias en extremidades inferiores desde hace 3 semanas, por lo que se automedicó vitamina B1, B6, diclofenaco y ketorolaco, sin mejoría. Además, debido a la presencia de fiebre, ardor al orinar, orina turbia y en poca cantidad en los últimos 5 días, se automedicó amikacina intramuscular 500 mg cada 12 h.

**Exploración física:** TA 160/100 mmHg, peso actual 80 kg, talla 1.54 m, índice de masa corporal 33.7 Kg/m^2^, circunferencia de cintura de 105 cm, FC 78 x minuto. Los hallazgos positivos fueron: sensibilidad disminuida en extremidades inferiores y edema pretibial +/++++. El resto de la exploración fue normal.

***Resultados de laboratorio:***

- Glucosa 141 mg/dL, Hemoglobina glucosilada 8%
- Creatinina sérica 1.3 mg/dL
- Colesterol total 162 mg/dL, HDL 58 mg/dL, LDL 95 mg/dL, Triglicéridos 140 mg/dL
- Ácido úrico 8.6 mg/dL
- Potasio sérico 3.8 mEq/L
- Tasa de filtración glomerular por fórmula CKD EPI de 62 ml/min/1.73 m^2^
- En examen general de orina: densidad de 1.021, cristales de oxalato +, proteínas +++ y leucocitos 1-2 por campo. Excreción urinaria de albúmina/creatinina de 1051 mg/g

***Manejo del equipo de salud:*** El ***médico*** indicó incrementar glibenclamida a 5 mg cada 12 h (la tomaba cada 24 h); continuar con el uso de aspirina 150 mg cada 24 h, losartan 50 mg cada 12 h y diclofenaco 100 mg cada 12 h. La administración de amikacina y ketorolaco fue suspendida. Interconsulta inmediata con el nefrólogo y el neurólogo. También le pidió disminuir notablemente el consumo de sal y le señaló los riesgos de la automedicación, lo citó en 1 mes con resultados de examenes.

A la ***nutrióloga*** le dijo que lleva una dieta para diabético prescrita por un médico familiar hace 2 años, la prepara él mismo, incluye nopales, acelgas, clara de huevo, frijoles, jitomate, avena, pescado y pollo. No consume carnes rojas, plátano, fresas, ni sandía. En general, consume verduras 2 veces a la semana. Cocina con aceite vegetal de canola y endulza su leche con 2 sobres de canderel. Consume 3 L de agua al día. En el análisis dietético se encontró un consumo diario de 2,800 Kcal (35 kcal/kg peso actual), con una distribución de 15% de proteínas (1.3 g/kg de peso actual), 60% de hidratos de carbono (sin azúcares simples) y 25% de lípidos. Le fue indicado un plan de 2,300 Kcal (28 kcal/kg peso actual), bajo en purinas, sin modificación de la distribución de macronutrimentos; disminuir consumo de líquidos y evaluación nutricional en 3 meses.

La ***enfermera*** detectó que su consumo de alimentos parece ser excesivo. Es totalmente independiente para realizar sus actividades diarias; vive con su esposa y un hijo, quienes aparentemente le dan apoyo emocional, pero no le ayudan en la preparación de sus alimentos o en otras actividades de la vida diaria. Convive con vecinos y amigos a diario. No realiza actividad recreativa ni ejercicio por no tener tiempo. Duerme 7 horas diarias. Acude a sus citas mensuales y dice tener buena adherencia al tratamiento prescrito. Cuando le diagnosticaron DM e hipertensión, pensó en quitarse la vida, pero actualmente ya no tiene esos pensamientos. Conoce sobre sus enfermedades, complicaciones y cifras ideales de tensión arterial y glucemia; las mide ocasionalmente (solo a veces lo registra). Sabe que peso debería tener, así como el nombre y la correcta administración de sus medicamentos.

A la ***trabajadora social*** le comentó que le gustaría sentirse más contento, no está satisfecho consigo mismo, ni con su familia. Su ingreso mensual es de 9,150 pesos. Su casa es rentada y cuenta con todos los servicios públicos. Tiene electrodomésticos básicos y cuenta con computadora.

1. **EN ESTE PACIENTE SON FACTORES DE RIESGO PARA ERC:**
   1. ____ Edad.
   2. ____ Antecedente de retinopatía.
   3. ____ Ingesta de diclofenaco.
   4. ____ Ingestión de vitaminas B1 y B6.

1. **EL CUADRO DE ESTE PACIENTE, ES COMPATIBLE CON:**
   1. ____ Dislipidemia.
   2. ____ Microalbuminuria.
   3. ____ Proteinuria.
   4. ____ Ingestión excesiva de proteínas
   5. ____ Obesidad grado 1.

1. **EN ESTE PACIENTE SON DATOS A FAVOR DE ERC:**
   1. ____ Excreción urinaria de oxalatos.
   2. ____ Densidad urinaria.
2. **EN ESTE PACIENTE SON INDICADORES ÚTILES PARA PRECISAR EL DIAGNÓSTICO NUTRICIO:**
   1. ____ Índice de masa corporal.
   2. ____ Ingestión energética.
   3. ____ Tasa de filtración glomerular.
   4. ____ Leucocitos en orina.
3. **DE ACUERDO CON EL ESTADO ACTUAL DEL PACIENTE SON ACCIONES APROPIADAS QUE LA NUTRIÓLOGA HIZO:**
   1. ____ Evaluar su historial dietético
   2. ____ Disminuir el aporte de calorías.
   3. ____ Disminuir el aporte de líquidos.
   4. ____ Mantener la distribución previa de macronutrimentos.
4. **ERAN ACCIONES NECESARIAS PARA EL ADECUADO MANEJO NUTRICIO DE ESTE PACIENTE, QUE LA NUTRIÓLOGA NO REALIZÓ:**
   1. ____ Medir la presión arterial.
   2. ____ Evaluar la ingestión de sodio en la dieta.
   3. ____ Calcular el peso seco.
   4. ____ Disminuir el consumo de proteínas.
   5. ____ Restringir la ingestión de potasio.
   6. ____ Establecer tiempos de comida apropiados.
   7. ____ Recomendar una alimentación más variada.
   8. ____ Recomendar cambiar por aceite de maíz para guisar.
   9. ____ Prohibir el consumo de lácteos.
5. **FUERON ACCIONES INNECESARIAS PARA LA PACIENTE REALIZADAS POR EL PERSONAL DE SALUD:**
   1. ____ Disminuir el consumo de líquidos.
   2. ____ Disminuir el consumo de purinas.
6. **EN LA PRÓXIMA CONSULTA DE ESTE PACIENTE LA NUTRIÓLOGA DEBE:**
   1. ____ Disminuir el consumo de calcio.
   2. ____ Incrementar el consumo de salvado como fuente de fibra.
   3. ____ Sugerir al médico que solicite electrolitos séricos.

**CASO 3**

***Antecedentes personales:*** Mujer de 47 años, casada, maestra de educación primaria. La madre murió por hemorragia cerebral secundaria a hipertensión arterial. El padre tenía insuficiencia renal y murió de infarto al miocardio y un hermano también murió por infarto al miocardio a los 47 años. La paciente fumó por 13 años (hasta 10 cigarrillos al día), dejó de hacerlo hace 9 años. Nunca ha ingerido bebidas alcohólicas. Hace bicicleta estacionaria 30 minutos al día. Se conoce con hipertensión arterial desde hace 23 años y con dislipidemia desde hace 6 años. Estuvo hospitalizada hace 2 años por isquemia cerebral transitoria. En sus exámenes de hace un mes: creatinina sérica 0.6 mg/dL y albuminuria/creatinuria 195 mg/g. Terapia actual: enalapril 10 mg/día, bezafibrato 200 mg/día, cafiaspirina® 2 tabletas/día y pravastatina 20 mg/día.

En su cita mensual de control con su médico familiar refiere disnea de medianos esfuerzos; cefalea intensa, pulsátil, localizada en región frontal; dolor en ambas rodillas, tobillos y costado derecho, de 2 meses de evolución.

***Exploración física:*** TA 150/90 mmHg, peso 81.5 kg, talla 1.58 m, índice de masa corporal 32.7 kg/m^2^ y circunferencia de cintura 94 cm. Resto de exploración física normal.

***Resultados de laboratorio****:*

- Glucosa 93 mg/dL
- Colesterol total 279 mg/dL
- HDL 30 mg/dL
- LDL 160 mg/dL
- Triglicéridos 386 mg/dL
- Creatinina sérica 0.6 mg/dL
- tasa de filtración glomerular fórmula CKD-EPI 101 mL/min/1.73 m^2^
- Albúmina/creatinina en orina 215 mg/g
- Urianálisis normal.
- Electrolitos séricos normales

***Manejo del equipo de salud***: el ***médico*** indicó continuar con la misma dosis de enalapril y bezafibrato; incrementar la pravastatina a 40 mg; agregar hidroclorotiazida 25 mg y aspirina 100 mg, todas cada 24 h; suspendió la cafiaspirina®. Fue enviada a nutrición. Cita en 1 mes con resultados de laboratorio.

La paciente informó al ***nutriólogo*** que lleva una dieta reducida en sal y grasas su dieta diaria incluye vegetales, licuado verde una vez por semana, aguacate, tortillas, pan, pescados, jamón de pavo, poca carne roja, 1 refresco de cola *light* y frecuentemente sopas instantáneas. No le gusta el pollo, no consume azúcar, agrega sustituto de sal a sus platillos y cocina con aceite de oliva. Al día ingiere 1,900 Kcal (23 kcal/ kg peso actual), 10% de proteínas (0.6 g/kg peso actual), 75% de hidratos de carbono (sin azúcares) y 15% de lípidos, 3.5 g de sodio, 5.5 g de potasio, 23 g de fibra y 2.5 L de agua. Se le indicó un plan de alimentación de 1,650 Kcal (20 kcal/kg peso actual), con una distribución de 16% (0.8 g/kg peso actual) de proteínas, 54% de hidratos de carbono y 30% de grasas, así como incrementar el consumo de agua a 3.0 L; ingerir sólo pollo y pescado y se le prohibió consumir carne roja. Se citó para evaluación en 1 mes.

Al ***enfermero*** le informó que sí sabe cuánto debe pesar y cuáles son las cifras de presión arterial que debe tener y la monitorea regularmente. Dice tomar sus medicamentos tal como se lo indica el médico. Vive con su esposo, alcohólico, con quien no lleva una relación satisfactoria y al momento de la consulta constantemente agrede verbalmente a la paciente. Tiene 3 hijos (de 8, 10 y 11 años). Es autosuficiente en actividades de la vida cotidiana. Tiene convivencia con vecinos o amigos a diario, quienes le apoyan emocionalmente. Sus actividades recreativas incluyen ir al cine y comer fuera de casa 1 vez al mes. Cuando le diagnosticaron hipertensión arterial le recomendaron modificar conductas nutricionales y de estilo de vida, lo cual ha intentado hacer.

En la entrevista de ***trabajo social*** informó que su casa es propia y cuenta con todos los servicios. Su ingreso familiar mensual es de 10,000.00 pesos. Tiene problemas para solventar los gastos familiares ya que todos sus hijos estudian y su marido sólo aporta 2,000.00 pesos mensuales. Está sometida a mucho estrés en su trabajo y tiene dificultad para conciliar el sueño; duerme menos de 5 horas diariamente.

1. **EN ESTA PACIENTE SON FACTORES DE RIESGO PARA ERC:**
   1. ____ Edad.
   2. ____ Ingestión prolongada de cafiaspirina®.
   3. ____ Ingestión de bezafibrato.
   4. ____ Obesidad.

**B) EL CUADRO DE ESTA PACIENTE ES COMPATIBLE CON:**

- 1. ____ Ingestión deficiente en grasas.
  2. ____ Obesidad grado 2.
  3. ____ ERC estadio 3.
  4. ____ Microalbuminuria.

**C) EN ESTE PACIENTE SON DATOS A FAVOR DE ERC:**

- 1. ____ Nivel de creatinina sérica.
  2. ____ Tasa de filtración glomerular.

1. **EN ESTA PACIENTE SON INDICADORES ÚTILES PARA PRECISAR EL DIAGNÓSTICO NUTRICIO:**
   1. ____ Alcoholismo del cónyuge.
   2. ____ Consumo de anti-inflamatorios no esteroideos.
   3. ____ Circunferencia de cintura.
   4. ____ Cifras de lípidos séricos.
2. **DE ACUERDO CON EL ESTADO ACTUAL DEL PACIENTE SON ACCIONES APROPIADAS QUE EL NUTRIÓLOGO HIZO:**
   1. ____ Evaluar los alimentos de mayor consumo
   2. ____ Incrementar la proporción de proteínas en la dieta.
   3. ____ Incrementar el consumo de agua.
   4. ____ Eliminar el consumo de carne roja.
3. **ERAN ACCIONES NECESARIAS PARA EL ADECUADO MANEJO NUTRICIO DE ESTA PACIENTE, QUE EL NUTRIÓLOGO NO REALIZÓ:**
   1. ____ Mantener el consumo bajo de lípidos.
   2. ____ Eliminar el licuado verde.

1. **FUERON ACCIONES INNECESARIAS PARA LA PACIENTE REALIZADAS POR EL PERSONAL DE SALUD:**
2. ____ Disminuir el porcentaje de hidratos de carbono.
3. ____ Prohibir el consumo de carnes rojas.
4. **EN LA PRÓXIMA CONSULTA DE ESTE PACIENTE EL NUTRIÓLOGO DEBE:**
5. ____ Evaluar el tipo de bebidas ingeridas.
6. ____ Instrucciones para disminuir el potasio en las verduras con técnicas

de remojo y cocción de los alimentos.

**CASO 4**

**Antecedentes personales**: Hombre de 26 años, soltero, con preparatoria completa, es empleado de una ferretería. No fuma, no ingiere alcohol, ni drogas. Jugaba fútbol, pero lo ha abandonado al presentar fatiga desde hace 3 meses. Desde los 18 años de edad presenta cefalea universal casi cotidiana, exacerbada por estrés laboral; se ha automedicado frecuentemente diversos analgésicos desde hace 7 años. Hace 8 meses presentó un cuadro de cólico renoureteral derecho que fue considerado como litiasis urinaria por un médico general (sin documentar la presencia de lito), que desapareció con sintomáticos al cabo de 1 día; en esa ocasión le dijeron que tenía creatinina sérica de 1.3 mg/dL y que su presión era normal.

Acudió con su médico familiar debido a que en las últimas 2 semanas se han exacerbado la cefalea, la fatiga, la epigastralgia y ha aparecido edema blando en extremidades inferiores y párpados.

**Exploración física:** Se le encontró TA de 160/110 mmHg, peso 62.0 Kg, talla 1.68 m, IMC 22.0 Kg/m^2^ , circunferencia de cintura de 80 cm. Mucosas con adecuada hidratación y palidez moderada. Edema +/++++ de ambos tobillos. El resto de la exploración física normal.

***Resultados de laboratorio****:*

- Hemoglobina 9.0 g/dL
- Glucosa 86 mg/dL
- Colesterol total 203 mg/dL
- Triglicéridos 380 mg/dL
- Creatinina sérica 1.4 mg/dL
- Tasa de filtración glomerular por fómurla CKD-EPI 69 mL/min/1.73 m^2^
- Sangre oculta en heces ++, urianálisis con densidad urinaria 1.020, pH 7.0, esterasa negativa, proteínas ++, 20 eritrocitos x campo, 4 leucocitos x campo
- En una recolección de orina de 24 h se reportó proteínas de 1.5 g/día.
- Electrolitos séricos normales

***Manejo del equipo de salud****:* el ***médico*** le indicó metoprolol 100 mg/día, hidroclorotiazida 25 mg/día, bezafibrato 200 mg/día y continuar con su misma dieta. Le recomendó acudir con el urólogo y el gastroenterólogo; le dio cita dentro de 1 mes con exámenes.

A la ***nutrióloga*** le refirió que hace 3 meses pesaba 70 Kg (pérdida del 11%), desde entonces su apetito ha disminuido. Nunca le han prescrito una dieta específica; le gusta comer carnes rojas o mariscos casi a diario, ocasionalmente come frutas y verduras. Agrega sal extra a todos sus alimentos; ingiere regularmente alrededor de 2.0 L de agua al día además de infusiones de hierbas chinas que le recomendó un amigo. Ingesta diaria de 1,600 Kcal (26 kca/kg), con una distribución de 25% de proteínas (1.6 g/kg peso actual), 40% de hidratos de carbono (10% azúcares) y 35% de grasas (15% saturadas, 10% monoinsaturadas y 10% poli-insaturadas), 6.0 g de sodio y 10 g de fibra. Se indicó plan de alimentación de 2,000 Kcal (32 kcal/kg), con 10% de proteínas (0.8 g/kg peso actual), 55% de hidratos de carbono y 35% de grasas. Se recomendó disminuir el consumo diario de agua a 1 L, de grasas saturadas, carnes, mariscos y sal. Se citó a evaluación nutricional en 1 mes.

Al interrogatorio por la ***enfermera***, respondió que se considera buen trabajador y afectivo con sus compañeros de trabajo, con los que comparte actividades sociales, ir al cine o jugar futbol. Considera que tiene un buen estado de salud y casi nunca visita al médico. Su trabajo ha limitado sus actividades desde hace 3 meses, tiempo en el que se han exacerbado sus síntomas.

A la ***trabajadora social*** le refirió haber sido hijo único y que desde los 18 años de edad, después de que fallecieron sus padres, decidió vivir con la familia de un compañero de preparatoria, ya que tiene distanciamiento emocional con sus familiares más cercanos. Es independiente en su manutención; su ingreso mensual es de 8,500.00 pesos, aporta 2,000.00 pesos para su alimentación; del resto de su salario, una parte la ahorra y la otra la utiliza para sus gastos personales.

1. **EN ESTE PACIENTE SON FACTORES DE RIESGO PARA ERC:**
   1. ____ Antecedentes familiares.
   2. ____ Uso crónico de anti-inflamatorios no esteroideos.
   3. ____ Dieta rica en mariscos.

1. **EL CUADRO DE ESTE PACIENTE ES COMPATIBLE CON:**
   1. ____ Ingestión excesiva de líquidos.
   2. ____ Ingestión adecuada de proteínas.
   3. ____ Proteinuria.
   4. ____ Enfermedad renal crónica estadio 4.
2. **EN ESTE PACIENTE SON DATOS A FAVOR DE ERC:**
   1. ____ Proteinuria.
   2. ____ Leucocituria.
   3. ____ Esterasa negativa.
   4. ____ Índice de masa corporal.
3. **EN ESTA PACIENTE SON INDICADORES ÚTILES PARA PRECISAR EL DIAGNÓSTICO NUTRICIÓN:**
   1. ____ Frecuencia de consumo de alimentos de origen animal.
   2. ____ Niveles de lípidos séricos.
4. **DE ACUERDO CON EL ESTADO ACTUAL DEL PACIENTE SON ACCIONES APROPIADAS QUE LA NUTRIÓLOGA HIZO:**
   1. ____ Incrementar el consumo diario de calorías.
   2. ____ Aumentar el consumo de hidratos de carbono totales
   3. ____ Disminuir el consumo de grasas saturadas.
   4. ____ Disminuir el consumo de agua a 1 L al día.
5. **ERAN ACCIONES NECESARIAS PARA EL ADECUADO MANEJO NUTRICIO DE ESTE PACIENTE, QUE LA NUTRIÓLOGA NO REALIZÓ:**
   1. ____ Evaluar la disposición al cambio de conducta.
   2. ____Aumentar la ingestión proteica para compensar las pérdidas por la orina
   3. ____ Disminuir la cantidad de hidratos de carbono simples.
   4. ____ Incrementar el consumo de fibra.
   5. ____ Incrementar el consumo de verduras y frutas.
   6. ____ Restringir los alimentos ricos en potasio.
   7. ___ Recomendar el consumo mínimo de 300g de pescado al día para

aumentar la ingestión de omega 3.

- 1. ____ Recomendar la eliminación de hierbas chinas.

1. **FUERON ACCIONES INNECESARIAS PARA EL PACIENTE REALIZADAS POR EL PERSONAL DE SALUD:**
2. ____ Indicación del médico de continuar la misma dieta.
3. ____ Incrementar el consumo de hidratos de carbono.
4. ____ Reducir el consumo de proteínas.
5. ____ Disminuir el consumo de agua a 1L al día.
6. ____ Cita a nutrición en 1 mes.
7. **EN LA PRÓXIMA CONSULTA DE ESTE PACIENTE LA NUTRIÓLOGA DEBE:**
8. ____ Evaluar el apetito.
9. ____ Reducir los alimentos ricos en oxalatos.
10. ____ Prescribir 2 copas de vino tinto.
11. ____ Dar una sesión de relajación.
12. ____ Orientar sobre la selección de bebidas saludables.
